# Recent advances in inflammatory bowel disease 

**Published:** 2017

**Authors:** Beata Polewiczowska, David Aldulaimi

**Affiliations:** *Department of Gastroenterology, South Warwickshire Foundation Trust, UK *

*van Langenberg DR, Yelland GW, et al* **.** ***Cognitive impairment in Crohn’s disease is associated with systemic inflammation, symptom burden and sleep disturbance.*** *United European Gastroenterology Journal 2017; 5(4):579-587.*

Patients with inflammatory bowel disease may suffer from cognitive dysfunction in addition to symptoms of diarrhoea and abdominal pain. Common complaints include impaired concentration and clouding of thought and they usually affect Crohns patients. A cross-sectional observational study has been conducted in order to measure cognitive impairment in Crohns patients compared to healthy controls. 

In this study, 49 Crohns clinic patients and 31 healthy control patients were asked to complete surveys including clinical, demographic, psychiatric, fatigue and sleep parameters. In addition, disease activity has been assessed for each Crohns patient using serum CRP, faecal calprotectin, Harvey-Bradshaw Index. A computer based test, The Subtle Cognitive Impairment Test (SCIT-RT) has been performed with the primary measure of response time and compared between both groups. Furthermore, multiple linear regression analyses incorporating all variables assessed for factors associated with slower SCIT-RT, indicating cognitive impairment (1).

Crohns patients had higher depression scores and poorer sleep quality compared with healthy controls. Response times (SCIT-RT) were significantly slower in Crohns patients compared to healthy control group. The slowed response times were significantly correlated with clinical indices of inflammation including the Harvey-Bradshaw Index and serum CRP. This confirms a strong link between Crohns disease related congnitive impairment and systemic inflammation. However, there was an inverse correlation between intestinal mucosal inflammation and SCIT-RT as indicated by inverse correlation of faecal calprotectin and cognitive function. Poorer sleep quality remains strongly associated with cognitive dysfunction. Furthermore, this study reinforces that Crohns disease is a multisystemic condition with wide range of signs and symptoms (1). 

*Bahrehmand F, Vaisi-Raygani A, et al* **.** ***Whole-Blood thiopurine S-methyltransferase genotype and phenotype concordance in Iranian Kurdish Ulcerative Colitis (UC) patients.*** *Clin Lab 2017; 63(5):947-954***.**

Thiopurine methyl trasferase (TPMT) level, a drug metabolizing enzyme, is routinely checked prior to treatment of Inflammatory Bowel Disease patients with thiopurine compounds (2). Caution should be used in homozygous recessive patients or individuals with very low TPMT activity due to concern for leukopaenia. An Iranian study looked at TPMT phenotypes and genotypes in IBD patients to predict the risk of TPMT toxicity prior to thiopurine treatment. Total of 210 ulcerative colitis patients and 212 healthy controls from Western Iran participated in this study. TPMT phenotype and genotype were determined by high performance liquid chromatography (HPLC), allele specific polymerase chain reaction (PCR) and restriction fragment length polymorphism (RFLP). The results indicated 2.2% of low TPMT activity in this populaton, no patients with TPMT deficiency and 97.8% of normal TPMT activity. This indicates low risk of thiopurine drug toxicity in IBD patients from Western Iran (2).

*Koller T, Galambosova M, et al* **.** ***Drug induced liver injury in inflammatory bowel disease: 1-year prospective observational study.*** *World Journal of Gastroenterology 2017; 23(22):4102-4111.*

Inflammatory bowel disease (IBD) treatment has significantly changed over the past decades. With the advance in new immunosuppression therapy, concerns have been raised about hepatotoxicity resulting from IBD management. Previous studies reported azathioprine and infliximab among five most common drugs causing liver injury (3). One study looked at drug induced liver injury in inflammatory bowel disease patients. Total of 251 patients were included in this prospective observational study which was carried out in a single IBD center. Aminotransferase and gamma-glutamyl transpeptidase levels were measured at baseline, 3 months prior to study entry and prospectively every 3 months for a year. Liver injury was classified as Grade 1 if ALT was raised up to 3 times the upper limit of normal, Grade 2 if ALT was raised more than 3 times the upper limit of normal, Hepatocellular injury if ALT was raised more than twice the upper limit of normal and Cholestatic injury in simultaneous GGT and ALP elevation above the upper limit of normal. 

Grade 1 injury was found in 66 patients (26.3%), grade 2 injury in 5 patients (2%), hepatocellular injury in 16 patients (6.4%) and cholestasis in 11 cases (4.4%). Infliximab and azathioprine were confirmed risk factors for liver injury. Patients on monotherapy with infliximab were observed to be at risk for hepatocellular injury whereas patients on azathioprine treatment were at higher risk of cholestasis. Although the liver injury was frequent, it did not change IBD management in most cases. This study also showed that increased BMI, fatty liver, monotherapy with infliximab and longer duration of therapy were all risk factors for hepatocellular injury (3). Liver injury in IBD patients in this study in most cases resolved spontaneously without change to disease management. This study suggests regular monitoring of liver function tests in all IBD patients on treatment. 

*Udden N SM, Peng L, et al* ***. NOD2 supressess colorectal tumorigenesis via downregulation of the TLR pathways.*** *Cell Reports 2017; 19:2756-2770.*

Inflammatory bowel disease is a major risk factor for colorectal cancer. NOD2 is the major inflammatory bowel disease susceptibility gene with 15%-20% IBD patients carrying mutations in NOD2. One study showed that deficiency of NOD2 leads to increased tumorigenesis in mice, which is independent of dysbiosis (4). NOD2 plays a critical role in the suppression of inflammation and tumorigenesis in the colon via downregulation of the TLR signaling pathways (4).

*Umar S, Clarke K, et al* ***. Diagnostic yield from colon biopsies in patients with inflammatory bowel disease and suspected cytomegalovirus infection: is it worth it?*** *Annals of Gastroenterology 2017; 30:429-432.*

Patients with inflammatory bowel disease are susceptible to infections due to immunosuppression. This includes a risk of reactivation of latent Cytomegalovirus infection (CMV). Current clinical practice includes CMV testing in acute flare of IBD patients who are refractory to treatment. A retrospective study was conducted in the United States to review IBD patients with suspected CMV infection. Pathology results of colonic biopsies of patients tested for CMV were reviewed over 3 year period. Positive CMV test result was considered based on positive hematoxylin and eosin staining and immunohistochemistry from two or more levels of a biopsy sample (5). 

Total of 125 patients tested positive for CMV according to these criteria, 99 of them (79.2%) were IBD patients (30 with Crohns disease, 63 had UC and 6 had indeterminate colitis). The results indicate that only one of 99 IBD patients had CMV colitis proven on colonic biopsy. 

Five patients out of the total 125 also tested positive for CMV, however none of them had IBD. One was post renal transplant, two patients suffered from haematological malignancies, one had graft versus host disease and one had ischaemic colitis (5). 

This study suggests that CMV infection in IBD patients is very rare despite the risk of immunosuppression. However, CMV infection has been recognized in 

post-transplant patients, haematological malignancies, graft versus host disease and ischaemic colitis (5).

*Qiu X, Ma J, et al* ***.*** ***Chemopreventive effects of 5-aminosalicylic acid on inflammatory bowel disease-associated colorectal cancer and dysplasia: a systematic review with meta-analysis.*** *Oncotarget 2017; 8(1):1031-1045.*

The effect of 5-aminosalicylic acid (5-ASA) on prevention of dysplasia and colorectal cancer in inflammatory bowel disease patients has been widely studied. A systemic review of literature and meta-analysis confirms that 5-ASA shows chemopreventive effect against colorectal cancer in IBD patients (6). However, this effect was observed in patients in ulcerative colitis and not in Crohns disease. Furthermore, mesalazine dose greater than 1.2 g per day showed greater protective effects against dysplasia and colorectal cancer than lower doses. However, treatment with Sulphasalazine did not show any benefit and protection regardless of the administered dosage (6).

